# Conservative Treatment of Ewing's Sarcoma of the Uterus in Young Women

**DOI:** 10.1155/2015/871821

**Published:** 2015-04-15

**Authors:** Giuseppe Loverro, Leonardo Resta, Edoardo Di Naro, Anna Maria Caringella, Salvatore Andrea Mastrolia, Mario Vicino, Massimo Tartagni, Luca Maria Schonauer

**Affiliations:** ^1^Department of Obstetrics and Gynecology, University Hospital Policlinico of Bari and University of Bari “Aldo Moro”, School of Medicine, Piazza Giulio Cesare 11, 70124 Bari, Italy; ^2^Department of Pathology, University Hospital Policlinico of Bari and University of Bari “Aldo Moro”, Piazza Giulio Cesare 11, 70124 Bari, Italy

## Abstract

Ewing sarcoma-primitive neuroectodermal tumors (ES/PNETs) constitute a family of neoplasms characterized by a continuum of neuroectodermal differentiations. ES/PNET of the uterus is rare. There are 48 cases of ES/PNET of the uterus published in the literature as far as we know. We describe a case of Ewing sarcoma of the uterus occurring in a 17-year-old woman presenting with a two-month history of pelvic pain. After surgical excision and microscopic, immunohistochemical, and electron microscopy examination, the diagnosis of Ewing sarcoma of the uterus was suggested. This report will discuss the diagnosis and surgical and clinical management of Ewing uterine sarcoma in young women, according to the available literature. In spite of the rarity of ES/PNETs, they should be taken into account in the differential diagnosis of uterine neoplasms in young women.

## 1. Introduction

Ewing sarcoma-primitive neuroectodermal tumor (ES/PNET) constitutes a family of neoplasms characterized by a continuum of neuroectodermal differentiations and by translocations involving EWS-FLI1 genes in approximately 85% of all cases. The term Ewing sarcoma has been used for undifferentiated tumors without evidence of neuroectodermal differentiation, as assessed by light microscopy and immunohistochemistry. The term pPNET has been used for tumors with apparent neuroectodermal features [1, 2].

Embryonal rhabdomyosarcoma (20%), Ewing sarcoma/primitive neuroectodermal tumor (17%), angiosarcoma (14%), and pleomorphic rhabdomyosarcoma (13%) appeared to be more common than the other sarcomas, although there was no single overwhelmingly prevalent histotype in the group. Almost all tumors of the Ewing sarcoma/PNET group have some form of EWSR1 (Ewing sarcoma breakpoint region 1) gene rearrangement, which is specific for this group of tumors.

Ewing sarcoma rarely occurs in the female genital tract and, until now, only 48 cases have been reported in the literature. Therefore, there is still a lack of accumulated experience with these histotypes due to different diagnostic and therapeutic approach.

Those pending problems are mainly important in cases occurring in very young women, when fertility sparing surgery is mandatory, in absence of life threatening conditions. This strategy has not been described until now.

## 2. Case Report

A seventeen-year-old nulliparous unmarried patient, referring menarche at 12 years, was admitted to our Institution with a diagnosis of abdominal mass and pelvic pain. Personal history evidenced irregular menstrual cycles and family history of breast and bladder cancer.

**Figure 1:**
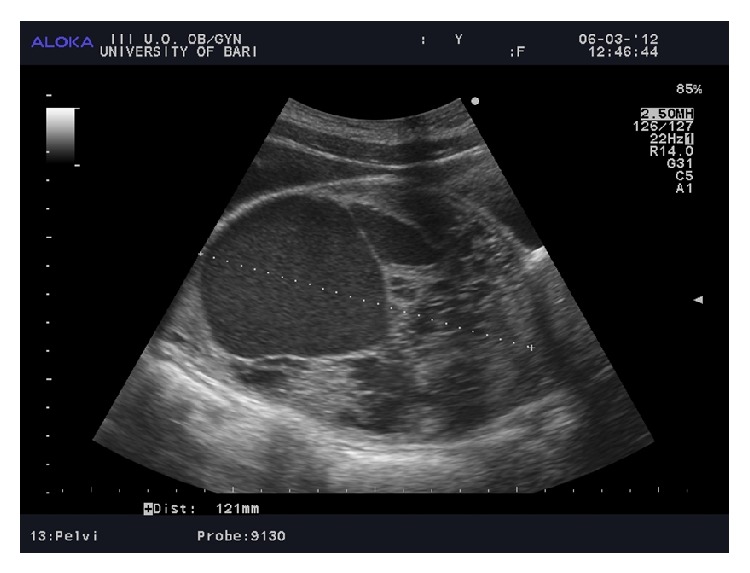
Transabdominal ultrasound showing a right parauterine mass with a complex echogenicity, 12 cm in diameter, deviating the uterus to the left side.

Transabdominal ultrasound revealed the presence of the right parauterine mass with a complex echogenicity, 12 cm in diameter, deviating the uterus to the left side (Figure 1). Serum tumor markers were negative.

The abdominal pelvis CT scan evidenced a parauterine right mass approximately 11 cm in diameter with a complex morphology, which contained some large, fluid filled, and hypodense areas, delimitated by dense septa and a pseudocapsule.

The right ureter appeared compressed by the mass, resulting in right ipsilateral hydronephrosis. Lymphadenopathies were absent.

Vaginoscopy and hysteroscopy excluded any vaginal wall or uterine cavity involvement, while the right fornix appeared convexed, due to overwhelming compression.

**Figure 2:**
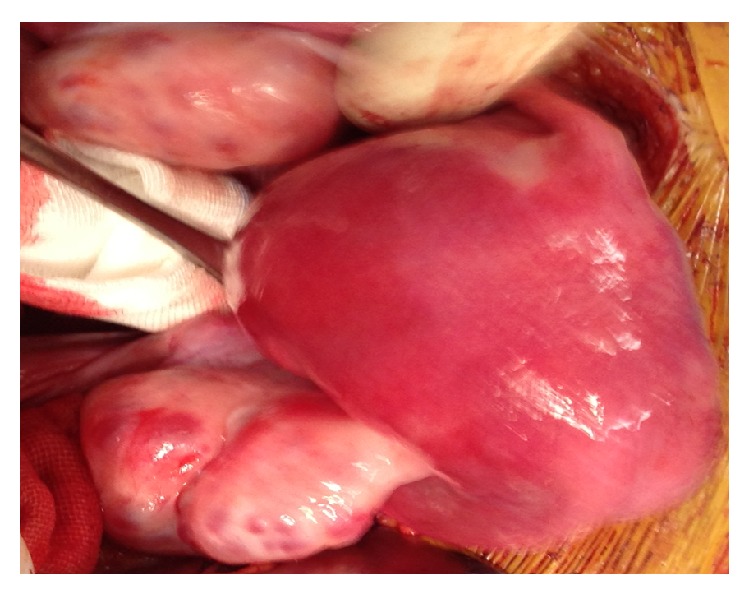
Laparotomy. A clear cleavage plane allows the dissection of the uterine mass from the underlying uterine myometrium. Anteriorly, the mass is dissected from the bladder and the right ureter, to the vaginal wall of the lateral right fornix.

A preliminary laparoscopy evidenced a 10 cm tumor arising from the anterior wall and right lateral border of the uterus, resembling a subserous fibroma, extending for 2 cm to the cervix and anterior vaginal wall (Figure 2).

At laparotomy, a clear cleavage plane allowed a dissection of the uterine mass from underlying uterine myometrium. Anteriorly, the mass was dissected from the bladder and the right ureter, to the vaginal wall of the lateral right fornix.

Intraoperatory histological examination was suggestive of mesenchymal neoplasm characterized by leiomuscular structures, with infiltration in the serosal layer by rounded cellular elements with little eosinophilic cytoplasm and dysmorphic large nucleus.

At the end of the excision, the lateral margin of the uterus was reconstructed along with the same technique of myomectomy.

**Figure 3:**
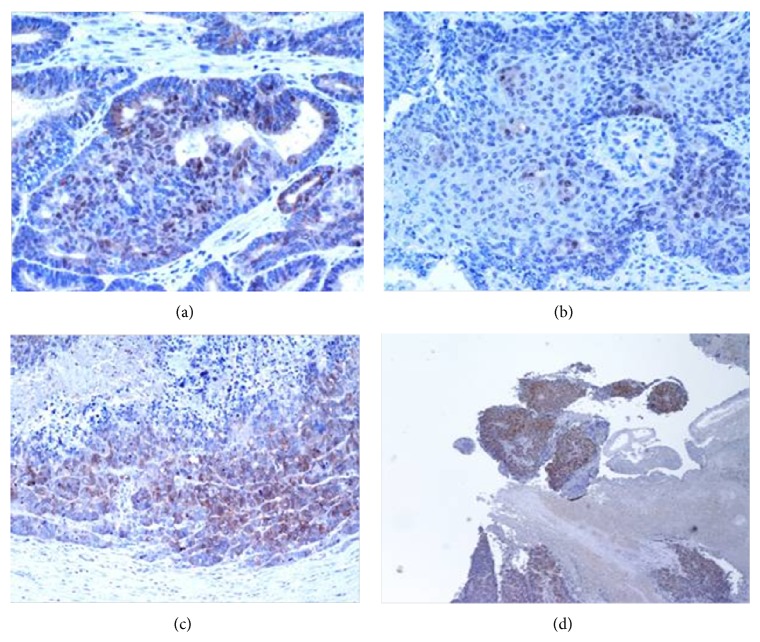
Poorly differentiated malignant neoplasm at histology: large cells, with scanty cytoplasm, hyperchromatic nuclei (a), uncohesive, diffusely dispersed, and occasionally disposed in tubular structures (c). The tumor shows being mostly necrotic with vital cells clasped around vessels (b). Immune-staining revealing a positive reaction for vimentin (+++) and CD99 (+++) (d). Proliferative index Ki67 is approximately 90%.

Final histological examination diagnosed a poorly differentiated malignant neoplasm. The cells were large, with scanty cytoplasm, hyperchromatic nuclei (Figure 3(a)), uncohesive, diffusely dispersed, and occasionally disposed in tubular structures (Figure 3(c)). The large part of the tumor was necrotic and the vital cells were clasped around vessels (Figure 3(b)).

Immunostaining revealed a positive reaction for vimentin (+++) and CD99 (+++) (Figure 3(d)) and an evidence for desmin, actin, ML, and CD10. The proliferative index Ki67 was approximately 90% (Figure 3). Morphological data and immunohistochemical studies were suggestive of extra skeletal Ewing's sarcoma.

The patient underwent chemotherapy (IVADo) and radiotherapy in the Protocol E*p*SSG RMS2005 (European Soft Tissue Sarcoma Committee) in the Pediatric Unit and is still PFS at two-year distance.

Follow-up at the end of the second year revealed a normal PET-CT and ultrasound evaluation without evidence of any abnormality in uterine and ovarian morphology.

## 3. Discussion

Primitive neuroectodermal tumors of uterus (PNETs) are extremely rare, mainly in female genital system. Few data are available concerning diagnostic procedures, prognosis, treatment, and follow-up modalities.

Ewing's sarcoma-primitive may occur rarely in the ovary, vulva, vagina, cervix, and uterus [3–5].

Until now about 48 uterine cases have been reported, though referred to as different terminology, such as Ewing sarcoma, peripheral PNET (pPNET), PNET (not otherwise specified), and uterine tumors with neuroendocrine differentiation.

Therefore, first at all, there is a need for common nosological standardization.

Ewing's sarcoma family tumour is a group of small round cell tumors, characterized by the specific translocation t(11;22)(q24;q12) and the specific transcript FLI1/EWS (and by the less common subvariants that involve chromosome 22 and EWS gene, with chromosome 21, 17, or 7).

The two most common entities are classic Ewing's sarcoma and the peripheral primitive neuroectodermal tumour (pPNET): most of the cases arise from the skeleton (and are often characterized by large soft part involvement); however in some cases (less than 20%) these tumors do not involve bone structures, arising from soft tissues.

Extraskeletal tumors are most commonly found along the central axis in the paravertebral location, particularly in the chest wall. However, these tumors can arise in any soft tissues and rarely in some organs including kidneys and lungs [1].

In female genital tract, these tumors can be found in vulva, vagina, cervix, and, most commonly, the ovary, while the uterine localization is very rare.

Uterine ES-PNET has a bimodal age distribution occurring in postmenopausal age or in adolescence, where fertility sparing surgery could be extremely important.

Since most cases (over 75%) of Ewing sarcoma occur in the postmenopausal period [2], a very limited experience is available on diagnosis and treatment of cases occurring in very young women.

Clinically, abnormal vaginal bleeding was the most common presenting complaint. Some patients had an enlarged uterus or a palpable pelvic mass, as in our case.

Histologically, most cases of Ewing sarcoma/PNET consisted of uniform small round cells with round nuclei containing fine chromatin, scanty cytoplasm, and indistinct cellular borders. However, some tumors show nuclear atypias or are composed of larger cells with vesicular nuclei and prominent nucleoli. In some cases, Flexner-Wintersteiner and Homer-Wright rosettes can be found and rare tumors show glial or ependymal differentiation [3].

Although well delimited, histological examination of our Ewing sarcoma revealed poorly differentiated malignant neoplasm, with only occasional rosettes.

The diagnosis of ES/PNET is based on histopathologic characteristics supported by the immunohistochemical features.

The differential diagnosis of Ewing's sarcoma/PNET requires confirmation by ancillary methods including immunohistochemistry and analysis of EWSR1 gene rearrangement.

Immunohistochemically, these tumors usually exhibit positivity for CD99, vimentin, and FLI-1 and in some cases focal positivity for cytokeratins [4].

Therefore diagnosis of Ewing sarcoma, pPNET or PNET (NOS), should be based on analysis of EWSR1 gene rearrangement or immunohistochemical analysis of FLI-1 not only on histological findings, associated with a limited immunophenotype usually represented by expression of CD99 [5].

Diagnosis of Ewing sarcoma should be based on positive immunohistochemical reactions for vimentin (+++) and CD99 (+++) and negative reaction for desmin, actin, ML, and CD10, associated as in our experience with a high (90%) proliferative index Ki67.

In fact, membranous expression of CD99 is sensitive but not specific for Ewing sarcoma since it can be found in lymphoblastic lymphoma, alveolar rhabdomyosarcoma, synovial sarcoma, some carcinomas (including endometrioid and serous adenocarcinoma), and many others [6].

As clinical point of view, serum markers are not pathognomonic, while fine needle aspiration cytology has been successfully adopted in few cases.

Imaging techniques are emerging diagnostic tool, although they have no well-established role, mainly due to the rarity of the disease.

Computed tomography imaging reports are not specific for the uterine Ewing sarcoma, as in other uterine sarcomas.

In a similar way, ultrasound still does not provide definitive diagnostic imaging. In our case, Ewing sarcoma appeared as a right large dishomogeneous parauterine mass, consisting of various cystic structures filled by hypodense fluid and delimitated by septa, extending downward to the right paravaginal space. These unusual findings, in a very young girl, could be suggestive of botryoid sarcoma, vaginal clear cells carcinoma, and yolk sac tumor. Hysteroscopy and CT may have a role in preoperative staging of advanced cases.

Up to date, established surgical approach has been simple or radical total hysterectomy. Moreover, sixteen patients underwent salpingo-oophorectomy and nine patients underwent lymphadenectomy [1].

In all three cases of very young girls, aged less than 18 years, simple or radical hysterectomy has been performed, on the basis of huge extension of the disease.

The present case is the first fertility sparing surgery, with tumor exeresis and myometrial reconstruction, in spite of an intraoperative histological frozen section diagnosis of FIGO stage I “mesenchymal tumor,” extending downwards to cervix and expanding in abdominal cavity, without any involvement of nearest structures.

However, cases with tumor confined to the uterine corpus had a significantly better result than counterparts and long-term survival has been reported in four cases diagnosed at an early stage, although in all cases total hysterectomy has been performed. Exceptionally, one patient with stage IIIc disease survived 10 years after treatment without evidence of disease recurrence.

Undoubtedly, presence of tumor pseudocapsule favoring a bloodless surgical exeresis and normal appearing of surrounding tissues is important for fertility sparing surgery in Ewing sarcoma occurring in a very young patient, mainly at initial stage.

Although only a better comprehension of the biology of uterine ES/PNETs could enlighten many aspects of clinical behavior [3], any aggressive surgical treatment in the above described conditions could be uneventful in the long-term PFS and overall survival, unless the uterus is clearly and totally involved. Therefore, in absence of different data, it seems that radical surgery is dictated by advanced stage more than intrinsic aggressiveness of the disease.

Moreover, conservative surgical option in early invasive Ewing sarcoma is mandatory in absence of massive local invasion or lymph node metastasis [2, 7] and is in agreement with pediatric treatment guidelines [7] and reflects prognostic factor of uterine ES/PNETs.

Conservative modality treatment is also encouraged by the multimodality therapy which has improved the prognosis of Ewing's sarcoma family tumors, allowing a 5-year survival in 60% of cases.

Response to chemotherapy is observed in more than 80% of cases, representing an important prediction factor of outcome.

Intensive chemotherapy regimens include alkylating agents (cyclophosphamide or ifosfamide), vincristine, actinomycin-D, and in most cases doxorubicin.

Recent studies suggest that doxorubicin, etoposide, and ifosfamide should be added to the classic cyclophosphamide-vincristine-actinomycin regimen; in detail, the North American randomized trial on Ewing's sarcoma and pPNET of the bone, reported by Grier et al. in 2003, described 5-year event-free survival and overall survival of 54% and 61% for patients treated with VACA regimen versus 69% and 72% for patients treated with VACA plus etoposide/ifosfamide, respectively [8].

Other findings would suggest a possible benefit for high-dose chemotherapy (with stem-cell rescue) in localized Ewing's sarcoma with unfavorable features (high tumor volume, axial site, and, in particular, poor response to induction chemotherapy).

On the other hand, other data would suggest that the behavior of extraosseous Ewing's sarcoma might be more similar to that of rhabdomyosarcomas: the historical series from the Intergroup Rhabdomyosarcoma Study (IRS) showed satisfying results with VAC regimen and no advantage with the addition of anthracyclines.

In conclusion, primary uterine ES-PNET is a rare and aggressive malignancy that requires early diagnosis and multimodal treatment.

In the context of the rarity of primary uterine Ewing sarcoma, we report the first case of fertility sparing surgery in an adolescent patient, completed with adjuvant chemotherapy, after comprehensive diagnosis, with a complete progression free survival at two-year distance.

In spite of the rarity, ES/PNET should be considered in the differential diagnosis of small cells neoplasms of the uterus.

Of course, to clarify the biological behavior of ES/PNETs in uterus, identification of more cases may prove to be beneficial.

Whenever possible, fertility sparing surgery is mandatory in young women, if it is possible to restore, after surgery, a normally functioning uterus, able to cope with a future pregnancy.
